# Membrane-Active Peptides and Their Potential Biomedical Application

**DOI:** 10.3390/pharmaceutics15082091

**Published:** 2023-08-06

**Authors:** Andreea Gostaviceanu, Simona Gavrilaş, Lucian Copolovici, Dana Maria Copolovici

**Affiliations:** 1 Faculty of Food Engineering, Tourism and Environmental Protection, and Institute for Research, Development and Innovation in Technical and Natural Sciences, Aurel Vlaicu University, Elena Drăgoi St., No. 2, 310330 Arad, Romania; andreea.andreea010@yahoo.com (A.G.); simona2213@yahoo.com (S.G.); lucian.copolovici@uav.ro (L.C.); 2 Biomedical Sciences Doctoral School, University of Oradea, University St., No. 1, 410087 Oradea, Romania

**Keywords:** membrane-active peptides, antimicrobial peptides, cell-penetrating peptides, tumor-homing peptides, peptide synthesis, cancer

## Abstract

Membrane-active peptides (MAPs) possess unique properties that make them valuable tools for studying membrane structure and function and promising candidates for therapeutic applications. This review paper provides an overview of the fundamental aspects of MAPs, focusing on their membrane interaction mechanisms and potential applications. MAPs exhibit various structural features, including amphipathic structures and specific amino acid residues, enabling selective interaction with multiple membranes. Their mechanisms of action involve disrupting lipid bilayers through different pathways, depending on peptide properties and membrane composition. The therapeutic potential of MAPs is significant. They have demonstrated antimicrobial activity against bacteria and fungi, making them promising alternatives to conventional antibiotics. MAPs can selectively target cancer cells and induce apoptosis, opening new avenues in cancer therapeutics. Additionally, MAPs serve as drug delivery vectors, facilitating the transport of therapeutic cargoes across cell membranes. They represent a fascinating class of biomolecules with significant potential in basic research and clinical applications. Understanding their mechanisms of action and designing peptides with enhanced selectivity and efficacy will further expand their utility in diverse fields. Exploring MAPs holds promise for developing novel therapeutic strategies against infections, cancer, and drug delivery challenges.

## 1. Introduction

Peptides perform numerous physiological activities, including membrane action as lytic agents or drug carriers. They can compromise the stability of lipid membranes by forming intricate molecular assemblies with them. Complicated molecular mechanisms heavily rely on the membrane’s lipid composition and the cell’s metabolic activities. As a result, these peptides have the potential to be effective for drug discovery and delivery, prompting research into their function and interactions with cell membranes [1,2,3].

The composition of cell membranes is highly diverse. In case of severe environmental change, organisms may even adapt by altering the makeup of their membranes in response to the significant changes [4]. For instance, bacteria can synthesize more lipids containing unsaturated acyl chains at lower temperatures. Therefore, designing novel peptides with the best possible cellular activity requires knowledge of the peptide structure, membrane composition, and environment [3]. Numerous antimicrobial peptides have been studied in various environments to determine the structure–function relationship that controls these peptides’ membrane activity. The studies have utilized neutral or zwitterionic vesicles that mimic eukaryotic membranes and negatively charged vesicles that mimic Gram-positive and Gram-negative bacteria [1].

### 1.1. Membrane-Active Peptide Classes

Membrane-active peptides (MAPs) include families of antimicrobial peptides (AMPs) and cell-penetrating peptides (CPPs) [3]. These two families have long been researched independently, most likely because AMPs were discovered first, although the distinction between AMPs and CPPs is only sometimes clear. A few studies have, interestingly, begun to close the gap between AMPs and CPPs in recent years. There is evidence that some CPPs can also be AMPs and vice versa. This finding suggests that the characteristics of bacterial cell membrane permeabilization and mammalian cell membrane penetration may share the same peptide sequence. In light of this, it could combine a host cell entrance characteristic with an antibacterial activity, which is crucial for therapeutic features [4].

Additionally, shared traits between the two groups, such as amphipathicity or the harboring of arginine-rich regions, indicate the possibility of dual roles. A single mutation can occasionally change a peptide’s ability to penetrate cells and become an antibacterial agent [2]. It has been demonstrated, for instance, that pVEC and TP10, formerly described as cell-penetrating peptides, are also AMPs [4].

Another example is CPP penetratin’s antibacterial action, which its arginine level can influence. Similarly, boosting the CPP Pep-1′s cationic nature increases its antibacterial activity. Cationic antibacterial peptides (CAPs) and CPPs share many physicochemical characteristics, yet they serve two distinct purposes [2].

#### 1.1.1. Antimicrobial Peptides (AMPs)

AMPs are small peptides that can potentially be multipurpose medicinal agents, as they work well against various bacteria. They are referred to as “natural antibiotics” [5]. Since the late nineteenth century, we have been aware that natural antimicrobials exist, specifically that leucocytes, lymphatic tissue, and blood contain antimicrobial compounds. Investigations of *Hyalophora cecropia* moths and human phagocytes were the first to draw attention to the idea that low-molecular-weight antimicrobial proteins were crucial for immunity [6,7,8]. Moreover, de novo designed short AMPs with only tryptophan, leucine, and lysine residues were synthesized as early as 1992, and the effects of tryptophan residue placement and the sequence length on bacterial inhibition were studied. Additionally, this study successfully created peptide sequences that can form amphipathic helices utilizing only arginine and valine as template residues. The obtained peptides were efficient against both Gram-positive and Gram-negative bacteria [6,9]. However, it is widely acknowledged that most AMPs primarily target membranes, and their therapeutic applicability is restricted by their interaction with eukaryotic cell membranes, which can be hazardous to host cells. As a result, scientists are working on redesigning or creating new AMPs with great antimicrobial action and little cytotoxicity toward eukaryotic cells [10].

Of all AMPs, 75.65% come from various animal species, including amphibians, insects, mammals, and fish. The remaining AMPs, which form 13.5% and 8.53% of all AMPs, respectively, are derived mainly from plants and bacteria [5].

Bacteriocins are another name for bacterial AMPs. They differ significantly from eukaryotic AMPs in many ways, despite having a similar action method and other similar traits. Bacteriocins are more potent at lower concentrations than eukaryotic AMPs. Bacteriocins also only have a limited impact on a few species or genera. Gram-positive bacteria create two primary categories of bacteriocins: lantibiotics (I), which contain unmodified AMPs, and non-lantibiotics (II), which include thioether-based ring structures called lanthionine or methyllanthionine [11]. In addition to the well-known natural antibiotics vancomycin and daptomycin produced by various actinomycetes species, novel AMPs obtained from soil-derived *Actinomycetes* strains include pargamicins B, C, and D produced by *Amycolatopsis* sp. ML1hF4; ohmyungsamycins A and B isolated from *Streptomyces* sp.; and the lipopeptide arylomycin A6 [5].

Marine ecosystems are among the best places to find AMPs because low temperatures, high pressure, complete darkness, and high salinity typically distinguish them. As a result, it has been claimed that marine AMPs have a distinct structural makeup and are better at adapting to harsh environmental factors like high salinity [5]. These ecosystems produce various AMPs exhibiting novel sequences, structures, and antibiotic potential with diverse biomolecules. Now-known sources of bioactive peptides for novel potential therapies include the venoms of multiple animals, including cnidarians, echinoderms, mollusks, arthropods, and vertebrates [12]. Marine AMPs include clavanins, hedistin, piscidin, myxinidin, pleurocidin, styelin, histidine-rich chrysophsin, proline- and glycine-rich collagencin, and proline- and arginine-rich callinectin, defensins, and discodermin A [5]. The process of discovering new medications typically involves isolating peptides from natural sources. Native peptides have some disadvantages as medications due to their poor plasma stability and inability to pass through cell membranes due to the hydrophilicity built into their structures. In this sense, peptide chemistry is essential for creating stable, functional, and precise peptide sequences with potential medicinal applications. Therefore, current techniques include using D-enantiomers, altering side chain length, utilizing N-methylation or bulky residues, terminal capping, and cyclization [13].

Plants are also considered significant providers of AMPs, since they must protect themselves against microorganisms to survive. Several plant AMPs with potential and diverse bioactivities, particularly antibacterial, anti-yeast, and antifungal activities, have been described from subterranean and all aerial parts, including flowers, fruits, leaves, bark, and seeds [14]. The purothionin from wheat flour (*Triticum aestivum*) was the first plant AMP discovered [15]. Plant AMPs are typically categorized based on the length of the peptide chain and the quantity and position of cysteines that form disulfide linkages. Defensins, snakins, puroindolines, glycine-rich proteins, cyclotides, hevein-type proteins, thionins, knottins, and lipid transfer proteins are just a few of the plant-derived AMP groups that have been isolated, identified, and characterized [5].

Antimicrobial peptides from insects are crucial components of their humoral immune systems. Insect AMPs are created in body fat and kept in hemolymph by insects. Since the discovery of antimicrobial action in the hemolymph of pupae from the gigantic silk moths *Samia cynthia* and *Hyalophora cecropia* in 1974 and the purification of the first insect AMP (cecropin) from *H. cecropia pupae* in 1980 [7], a large number of insect AMPs have been purified or discovered [16]. To date, more than 200 AMPs from insects have been found. Cecropins, insect defensins, glycine-rich peptides, proline-rich peptides, and lysozymes are the five main classes into which these peptides are divided [5]. Analysis of genomic sequences can also identify orthologous AMP genes in different insect species. Insect AMPs are active against various microorganisms, including bacteria, fungi, certain parasites, and viruses [17]. Even AMPs from the same class but from various insect species may have different antimicrobial activities. This may be due to variations in the ability of AMPs from various insect species to bind to bacteria. The capacity of an AMP to attach to a microorganism and its conformational modification to a more helical structure determine whether the AMP is active or not against the pathogen. The antimicrobial activity of a single insect AMP may not be potent, but the combined antimicrobial activity of all the AMPs in the hemolymph may be strong and substantial [16].

The AMPs isolated from natural sources present several issues that prevent their extensive therapeutic application, such as low stability, limited salt tolerance, and high toxicity. Changes in the structural and physicochemical factors can impact the antimicrobial activity of peptides. A study examining the structure–activity relationship of AMPs demonstrated the connection between the physical, chemical, and structural characteristics and biological activities of naturally occurring peptides and peptides artificially created from scratch [5]. As a result, it was feasible to develop peptides with good stability and broad-spectrum action. By altering the sequences of the naturally occurring antimicrobial peptides from various animals, several techniques have been developed to create novel synthetic antimicrobial peptides. It has been proven that even slight variations in the proportion of amino acids can cause a peptide’s structural and physicochemical properties to change. Truncation, amino acid replacement, hybridization, and/or cyclization have typically been used to modify the template peptide [18]. In the large-scale manufacturing of synthetic AMPs, truncating the AMP sequence yields short peptides, which lowers production costs. Compared to linear peptides, cyclic AMPs have increased membrane permeability. Another successful method for generating synthetic peptides is hybridization. The different desirable features of template peptides can be used by mixing snippets from naturally occurring AMP sequences. For instance, the generation of new chimeric AMPs with high antimicrobial activity and low toxicity is made possible by combining AMPs that display low toxicity and activity with AMPs that demonstrate high activity but substantially higher toxicity. It is feasible to create peptides using de novo AMP design that are only loosely related to natural AMPs in terms of amino acid frequency and position [19]. Studies have demonstrated that the common occurrence of amino acid residues in AMP databases can be exploited to create peptides. Another strategy combines “peptide motifs from databases,” which contain residues that are often used. To design AMPs, various computer techniques, including genetic algorithms, machine learning techniques, and language models, were applied. These approaches incorporate crucial data on AMP sequence bioactivities and biochemical factors. As a result, a candidate sequence’s antibacterial potential can be predicted before synthesis [5].

#### 1.1.2. Cell-Penetrating Peptides (CPPs)

It is difficult for more significant macromolecules like peptides, proteins, or nucleic acids to cross lipid membranes. Numerous strategies have been developed to stimulate cells to ingest macromolecular compounds to overcome this restriction. Biological instruments include viral vectors and physical ways of transfecting cells, from electroporation to microinjections. Detergents or membrane-active peptides are examples of chemical compounds that enable cellular entrance [20]. CPPs, also referred to as protein-transduction domains (PTDs), are a type of membrane-active peptides that consist of 5–30 amino acids and can transport a variety of biomolecules into cells, such as small drugs, small interfering RNA, proteins, peptide–nucleic acid (PNA), imaging agents, and vaccines [21].

CPPs have a history that dates back to 1988 [22]. The first CPP with the ability to translocate across membranes was described as the human immunodeficiency virus transactivating transcription protein (HIV Tat) [23]. Then, in 1991, researchers identified the related protein Penetratin/Antp from the antennae of *Drosophila melanogaster* [24]. In 1997, researchers identified the amphipathic penetrating peptides MPG, truncated HIV-Tat, and VP22, a structural protein originating from the *Herpes simplex* virus. In 1998, a study that reports on the first chimeric peptide, transportan, was published [25]. Following that, SS-31, Pep-1, and Oligo R were all found one after another [22]. The delivery of bioactive molecules was then reported for other CPPs with various charges, polarities, and structural characteristics [21]. The same peptides frequently show diverse internalization pathways at varying temperatures and concentrations. CPPs’ capacity to enter cells under various conditions has been demonstrated. There have been reports of CPP internalization in more than 80 different kinds of cell lines [26]. CPPs can deliver therapeutic molecules (drugs or vaccines) into cellular compartments using covalent or non-covalent bonding. Additionally, when exposed to lower endosome pH levels, CPPs can change their characteristics and turn membrane lytic. The GALA peptide, which has glutamic acid residues, is one example [27].

Cell-penetrating peptides are categorized based on their origin, chemical charge (cationic, amphipathic, and hydrophobic groups relevant to their absorption process), physicochemical features (e.g., charge, hydrophobicity, and residue distribution in the peptide sequence), and the level of alterations and design efforts [21].

CPPs can be divided into three categories based on their origin: (I) protein-derived peptides (e.g., transactivator of transcription (Tat), penetratin), (II) chimeric peptides (e.g., transportan), and (III) synthetic peptides [26].

The vast majority of CPPs are produced from naturally occurring proteins and peptides, including viral proteins, heparin-binding proteins, DNA- and RNA-binding proteins, homeoproteins, signal peptides, plant circular skeletal proteins, AMPs, and transcription factors [2,28,29]. The development of CPPs has directly utilized these proteins as templates. The delivery of a wide variety of substrates, including proteins, antigens, and PNAs into the cytoplasm and cell nucleus, is possible with the obtained peptides because they exhibit no cell specificity. Tat, vascular endothelial cadherin-derived CPP (pVEC), and penetratin are examples of CPPs that are shortened copies of full-length proteins [2].

Chimeric CPPs are composed of two or more motifs from different peptides. Transportan is a chimeric CPP derived from galanin and mastoparan [30]. Chimeric peptides can be made by covalently connecting a CPP’s hydrophobic domain to a nuclear localizing signal, resulting in amphipathic CPPs [31].

Synthetic cationic peptides that contain homopolymers of arginine, lysine, and/or the cationic amino acid ornithine are effective transduction peptides. Even histidine, which is protonated at low pH levels, can work as a CPP at levels below 6.0 and has been utilized to deliver drugs to tumor cells [31]. The polyarginine family, which includes the simplest CPP mimicking arginine as the only structural component, is classified as a synthetic peptide [30]. Recent research shows that non-primary and synthetic amino acids can enhance the CPPs’ characteristics even more. For instance, switching lysine residues for ornithine can improve resistance to cellular breakdown [2].

Their physical and chemical characteristics indicate that CPPs can be divided into cationic, amphipathic, and hydrophobic groups. Short amino acid sequences known as cationic CPPs have a net positive charge at physiological pH. They are primarily made up of basic amino acids like arginine, lysine, and histidine. According to studies on the peptides’ uptake, the number and arrangement of the amino acids inside the peptide sequence are crucial for transport. Penetratin, HIV Tat, and the polyarginine family are a few examples of cationic CPPs [32]. Regarding amphipathic peptides, their hydrophilic (often cationic) and lipophilic sections are in charge of making the first contact and assisting translocation through the plasma membrane. Primary amphipathic, secondary amphipathic α-helical, β-sheet amphipathic, and proline-rich amphipathic CPPs are subcategories of amphipathic peptides. MAP, MPG, BAC 7, CADY, pVEC, and Pep-1 are examples of amphipathic CPPs. Hydrophobic peptides categorized in a third class include TP10, C105Y, Pep-7, and Bax-inhibiting peptide (BIP). Their net charge is low. For their internalization into the membrane core, their hydrophobic motifs are essential. Compared to cationic, amphipathic peptides, the number of hydrophobic peptides is constrained [2,29,31].

#### 1.1.3. Tumor-Homing Peptides

A particular type of CPPs, called tumor-homing peptides (THPs), has attracted great interest because of their ability to interact specifically with the tumor microenvironment. THPs are short peptides (3–15 amino acids) with common motifs like RGD and NGR, which specifically bind to a surface molecule on tumor vasculature or tumor cells [33]. Using in vivo phage display in metastatic mouse prostate cancer xenograft models, the first tumor-penetrating peptide, iRGD, was discovered. RGD peptides are a particular class of peptides that contain the Arg-Gly-Asp sequence, which is widely distributed throughout the body and serves as the recognition site for the interaction between integrins and their ligand proteins. The RGD integrin recognition motif-containing peptides can bind to αv integrins (αvβ3 and αvβ5), which are abundantly expressed in the tumor vasculature [34]. Targeting THPs to receptors that promote endocytosis and enable ingestion of the receptor-bound THPs and their cargo typically confers their ability to penetrate cells [35].

### 1.2. Methods of Obtaining Membrane-Active Peptides

**Figure 1:**
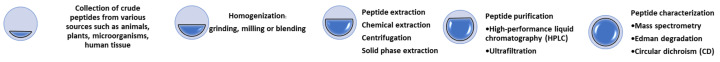
Pathways used to obtain membrane-active peptides from natural sources: isolation techniques, purification, and characterization [36,37].

There is no universal technique for isolating membrane-active peptides because of source and chemical composition heterogeneity. To obtain membrane-active peptides, various extraction and purification procedures have been used. Figure 1 illustrates the overall process [36]. These approaches are discussed further.

#### 1.2.1. Extraction of Membrane-Active Peptides from Natural Sources

Membrane-active peptides such as AMPs and CPPs are derived from naturally occurring living organisms like plants, frogs, insects, fungi, bacteria, and other organisms using several processes like extraction and purification. Although basic extraction techniques isolate peptides directly from source materials, more advanced techniques may occasionally purify them from crude extracts [5].

#### 1.2.2. Peptides Produced from Genetically Modified Organisms

Some restrictions are related to the conventional methods of AMP or CPP manufacturing. For instance, it is costly and time-consuming to purify AMPs from natural sources such as bacteria, plants, frogs, insects, or fungi [38]. Additionally, it might be challenging to produce AMPs with consistent activity and high purity, and each source needs to be purified using a different extraction technique. Peptide synthesis is only appropriate for small-scale production, such as laboratory applications, because the chemical synthesis of AMPs or CPPs is costly. As a result, the creation of these peptides through recombinant means, based on the expression of genes from natural sources in host organisms, has recently received increased interest. In addition, recombinant manufacturing may allow for peptide sequence alterations or the manufacture of wholly synthetic comparable peptides for various goals, such as enhancing peptide stability or creating hybrid peptides with potent antibacterial action. The expression of AMPs/CPPs has been achieved in different bacterial host cells. However, the most popular recombinant bioreactor is E. coli because of its well-known genetic, physiological, and biochemical characteristics, rapid growth, and rapid development [39]. Combining the antibacterial peptide with a carrier protein during the synthesis in bacterial hosts lessens the peptide’s deadly impact on the host organism and confers resistance to proteolytic destruction. Several recombinant AMPs, including cathelicidin LL-37, protegrin-1 (PG-1), dermsidin (DCD), ABP-CM4 peptide, LfcinB-W10 (a derivative of bovine lactoferricin), and various β-defensins have been generated in E. coli using the fusion protein approach. To boost the antibacterial activity of heterologous products and achieve a high yield, hybrids with various features have also been created and expressed by combining numerous AMP/CPP genes [5].

#### 1.2.3. Solid-Phase Peptide Synthesis

Solid-phase peptide synthesis produces AMPs and CPPs in laboratories and industrial settings. The main synthesis methods used are Fmoc-chemistry and Boc-chemistry [5].

The reaction conditions for the deprotection of the α-amino group of the final linked amino acid are the primary distinction between the Fmoc- and Boc-chemistry SPPS. In Fmoc-chemistry, the Fmoc group is removed using bases like piperidine to yield a free amino group immediately. However, in Boc-chemistry, the protonated ammonium species produced by the deprotection with TFA must be neutralized before the subsequent amino acid can be linked [37].

In Boc-chemistry, the deprotection of the amino group always takes place quantitatively, whereas, in Fmoc-chemistry, most of the detected complex couplings are preceded by challenging (slow, frequently incomplete) deprotection processes [37].

*Fmoc-chemistry synthesis:* During Fmoc-chemistry synthesis, the developing chain (peptide or oligomer) is stuck to a solid support like a resin or bead. The peptide synthesis commences at the C-terminus to reduce racemization. The carboxylic acid group of the additional amino acid and the amino-terminal group of the amino acid connected to the solid phase are selectively coupled using the “Fmoc strategy” to produce peptide growth [37]. Reagents are employed at high concentrations during the synthesis, and extra reagents can be easily removed by washing and filtering procedures after each binding step. Although this method can create peptides with fewer than 30 amino acids, larger peptides have only a 55% chance of having the target peptide’s proper sequence [5].

*Boc-chemistry synthesis:* The NH_2_-terminus of the resin-bound peptide is connected to the Boc-aminoethylsulfonylethyloxycarbonyl handle [38]. After that, the protocol for Boc-chemistry solid-phase peptide synthesis implies the removal of the Boc protecting group of the last coupled amino acid by brief treatment with 100% TFA (trifluoroacetic acid). The free amine that results from removing the Boc group can be changed with different functions for reactions with complementary groups on solid supports. [38]. After this, neutralization of the TfaO Æ +NH_3_-peptide-resin salt with DIEA (N, N-diisopropylethylamine) is carried out, and coupling of the next preactivated Boc-amino acid takes place. A thorough flow wash with DMF (dimethylformamide) is performed between these processes to eliminate extra reagents and reaction products that are not bonded to the resin. Researchers discovered that coupling yields for specific sequences dramatically increase when the second and third cycle steps are completed simultaneously [37]. A peptide functionalized for covalent chemo-selective interaction with the column support is produced by cleavage from the resin. The handle is cut into the desired full-length peptide using base treatment, which is then eluted from the column [38].

*Microwave-assisted synthesis.* Research describes a quick solid-phase peptide synthesis process using microwave assistance. The synthesis method is mainly based on pulsed microwave irradiation cycles with periodic reaction cooling during elimination of the Fmoc protective group and coupling. A suitable nonapeptide is synthesized using microwave technology with the best yield and purity. Furthermore, the approach applies to an extensive variety of peptides employing Boc-SPPS (solid-phase peptide synthesis), particularly Boc-SPPS of significant peptides by native chemical ligation. The automated microwave-assisted synthesis reduces the time required to assemble roughly 30-amino-acid peptide chains in an overnight process. It produces peptides with high yields and with excellent purity [40]. Obtaining short cycle times and crude peptide products with admissible purity (50–70% purity), which provide few challenges for HPLC purification, is the main advantage of microwave peptide synthesis [41].

#### 1.2.4. Supported Liquid Membranes for Peptide Extraction

According to studies, supported liquid membranes using Aliquat 336 as a carrier are used to extract small peptides. The extractions are conducted from an aqueous donor phase with a pH of 10 to a salt-containing acceptor phase. The gradient in salt concentration in between steps is what promotes mass transfer. The counter-ion type and concentration in the static acceptor phase, as well as the mass flow of the donor phase, affect the extraction efficiency. The concentration and configuration of the investigated peptides also impact the extraction efficiency [42].

### 1.3. Purification and Characterization of Membrane-Active Peptides

During the extraction process, AMPs are partially purified. After extraction, these highly purified extracts are subjected to various purification and characterization techniques, such as those listed above, to obtain maximum purification and structural identification [36].

#### 1.3.1. High-Performance Liquid Chromatography (HPLC)

HPLC is currently widely acknowledged as the most effective technology for assessing and purifying a broad spectrum of compounds. In general, polarity is used to classify amino acids. Likewise, the side chains’ size, form, and properties vary significantly within each group. Hence, the amount of charged groups and hydrophilicity/hydrophobicity are vital factors in peptide isolation [36].

Reversed-phase HPLC (RP-HPLC) is a widely utilized separation technology for AMPs, particularly amphibian AMPs [36]. As bacteriocins are typically resistant to the various organic solvents used as mobile phases and the high pressures applied during the chromatographic process, this analytical approach has been demonstrated to be highly beneficial for characterizing these antimicrobial peptides [37]. When the sample has been separated using cation exchange chromatography or solid-phase extraction chromatography, the bacteriocin extract is placed onto the HPLC column according to the HPLC methodology. The silica-based C4 to 18 ODS RP columns are the most common among the various RP column types used in the purification regimens [36].

#### 1.3.2. Ultrafiltration

By exerting a force of hydraulic pressure gradient via an appropriate membrane, ultrafiltration separates the solute from the solvent [43]. Because AMPs have a higher molecular weight than the solvent, it is typically utilized to isolate and concentrate AMPs (e.g., an antimicrobial peptide (NKT) from bovine hemoglobin hydrolysates [44]) from their solution [36].

#### 1.3.3. Mass Spectrometry

Mass spectrometry can identify, describe, and quantify the biomolecules. It is possible to calculate the molecular weight of polypeptides using standard mass spectrometry [36]. The effectiveness of mass spectrometry procedures has been improved by two advanced functions: Time-of-Flight (TOF) analyzers, which have also been used in association with Electrospray Ionization mass spectrometry (ESI-MS), and Matrix-Assisted Laser Desorption Ionization mass spectrometry (MALDI-MS) [45].

#### 1.3.4. Edman Degradation

Edman degradation is frequently used to investigate the primary structure and *N*-terminal configuration of AMPs [36]. Using a solid-phase peptide sequencer machine, peptides are successively cut on a solid support in this procedure. The resin-bound peptide is contained in a column that is pumped with reagents. The amino acid thiazolinone produced during each digestion cycle is gathered in a fraction collector for future investigation [46].

#### 1.3.5. Circular Dichroism (CD)

CD spectroscopy is the most common approach for determining the secondary structures of proteins and polypeptides in solution. This method can differentiate ordered (α-helix or β-sheet structures) and randomly oriented (random coil) structures. CD can identify wavelength-dependent changes by measuring the absorption of right- and left-circularly polarized light by optically active molecules like peptides and proteins [47].

### 1.4. Physicochemical Properties 

Antimicrobial peptides are typically between 10 and 50 amino acid residues long and have fewer than 60 amino acid residues. Additionally, they have an excess of basic lysine, arginine, and histidine residues, contributing to their positive net charge, typically between +2 and +9 (most frequently +4 to +6). Furthermore, they often include almost 50% hydrophobic residues. As a result, when these peptides interact with membranes, they frequently have spatially separated hydrophobic and hydrophilic regions and exhibit amphipathic features [48]. The physicochemical features of AMPs serve as the main determinants of the antibacterial potency of AMPs, and many approaches to the design of AMPs have been documented. The characteristics that have the most significant impact on the antibacterial activity of AMPs are net charge and hydrophobicity.

Moreover, specific amino acids are crucial in influencing both the antibacterial action and cell cytotoxicity of AMPs. With rising net charge and hydrophobicity, peptides become more capable of perforating microbial membranes and vice versa [49]. It also appears that net charge is a significant factor in the binding process, since peptides with more considerable positive charges have increased binding affinity with artificial membranes that are negatively charged [49]. Table 1 presents several membrane-active peptides, including antimicrobial peptides (AMPs) and cell-penetrating peptides (CPPs), focusing on their physicochemical characteristics and origin.

The capacity of AMPs to partition into the lipid bilayer of the microbial membrane is controlled by hydrophobicity. However, when hydrophobicity levels rise, mammalian cell toxicity and antimicrobial specificity loss become more prevalent [49].

**Figure 2:**
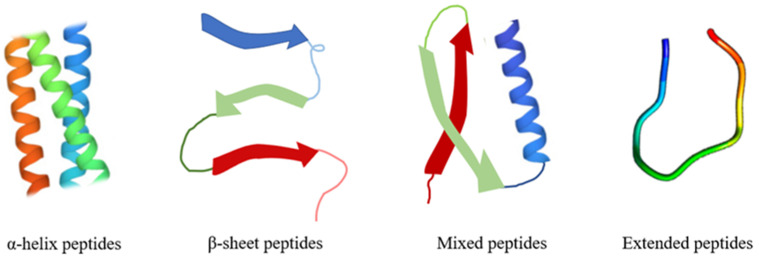
The 3D structures of different classes of AMPs, reconstructed after the original figure from [51]. Reproduced with permission.

Numerous short, linear antimicrobial peptides lack a folded structure when they are free in solution. They might, however, acquire shape after interacting with host cells by attaching to a particular receptor to start the biological reactions to microbial invasion [51]. Additionally, when these AMPs associate with bacterial targets like membranes, they may take on a specific shape. Nuclear magnetic resonance (NMR) spectroscopy using a multi-dimensional solution significantly influences the short peptides’ known 3D structures. Due to the structural stability of disulfide bonds, some AMPs have a folded structure in aqueous solutions. NMR or X-ray crystallographic techniques can be used to determine the structures of these tiny proteins. Similar structures are typically identified when both methods are used [52]. Based on their secondary structure, AMPs can be divided into four main categories: α-helix, β-Sheet, α/β, and extended/random-coil peptides (Figure 2). α-helix and β-Sheet are the most abundant in nature [53].

Most CPPs have a net positive charge at physiological pH, contain between 5 and 42 amino acids, and are partially hydrophobic, water-soluble, and/or polybasic in nature. They are rich in arginine and lysine residues [54]. Amphipathic (or amphiphilic) CPPs feature alternating areas of polar (hydrophilic) and non-polar (hydrophobic) amino acids in their structure. The resulting charge may be positive, neutral, or negative. Proline-rich peptides are a subcategory of amphipathic CPPs. The secondary structure of the peptide chain is broken by a proline residue that does not form a hydrogen bond with the nitrogen atom of the pyrrolidine ring. Hydrophobic CPPs contain a range of hydrophobic amino acid residues, including alanine, leucine, isoleucine, phenylalanine, tryptophan, methionine, and tyrosine [55].

CPPs vary in amino acid content and 3D structure, with cationic, anionic, and neutral sequences exhibiting different degrees of hydrophobicity and polarity. CPPs do, on average, exhibit sequence homology, which results in various absorption mechanisms and uptake levels. There are not many CPPs that contain structural data. Most of them presumptively adhere to a random coil. Increased helicity frequently enhances penetration. Only a few with secondary structure assignments have their 3D structures established [2].

## 2. Mechanisms of Action

### 2.1. Interactions of Membrane-Active Peptides with a Model or Biological Membranes

There are antimicrobial (AMP), amyloid (AP), and cell-penetrating peptides (CPP), which are categorized based on their direct mechanism of action manifested [56]. AMPs have significant medical importance due to their ability to act against various microorganisms. Generally, they follow two main routes: breaking up the cell membrane or determining cytoplasmic fluctuations [57]. Their main disadvantage is the significant level of intoxication they can cause [58]. APs are believed to determine a toxic response in the organism. The mechanism needs to be fully explained. The main points underline the possibility that some intermediary structural forms determine the immunological answer. Another case is the specific interaction with the membrane [59]. The research in this domain presents the biding mechanism, not affecting the bilayer, as possible for the non-toxic AP. CPPs belong to the small class of molecules that show an additional important characteristic: they can expand their action throughout transmembrane mechanisms. This feature qualifies them as excellent transfer vectors for different exogenous molecules. Their acting paths are diverse and depend on unique chemical characteristics. The strong positive type typically has more than eight positive charges and an endocytotic track. Depending on the amount in the reaction media, these can move into the membranes directly [60]. The behavior may be due to the absence of 3D structure. The second form of amphipathic CPPs acts appropriately, forming a partial helix. The breaking-through characteristic could depend on some recurrent amino acids on the water-repellent or water-soluble side. Independent of the specific action mechanism, all these present similarities regarding the fundamental physical mechanisms, mainly remodeling the phospholipid bilayer [56].

Even if the APs usually raise toxicological concerns, this aspect is not excluded in the case of the other peptides of interest. For this reason, the characteristic is also evaluated in different situations to increase the possibilities of using them in medical therapies. The piscidine *Of-Pis1*, a recently isolated peptide from rock bream (*Oplegnathus fasciatus*), proved its potential in the test against different pathogens [61]. *Of-Pis1* has a spiral arrangement only in the presence of an *Escherichia coli* membrane. It also presents activity with respect to the mammalian cell layers. It is vital to determine their optimum action parameters to obtain reliable results on peptides’ possible use in the pharmacological domain. Different investigations in this regard highlight this aspect [60,61,62]. Other elements, distinct in their impact on the cell membrane, influence permeability [63].

It has been shown that in the case of peptides designed for antifungal activity, the presence of ergosterol receptors facilitates their binding on the mycotic membrane. The interrelationship can be boosted further by phosphatidylethanolamine [64].

AMPs and CPPs are now incorporated into the MAPs family. A reference representative of this category is melittin. It has a linear cationic structure that was successfully used for studying patterns [65]. The most used methodologies to characterize them are based on fluorescence microscopy or spectroscopy, flow cytometry, fluorometry, biomembrane force probes, and photolabeling, all presenting specific limitations and advantages [4].

A current trend is to develop MAPs with more than one function. The Chim2 was intended to have, besides an antimicrobial effect, the capacity to release immunomodulatory chips. The mechanism targeted the possibility of directing the peptide adsorbed by the membrane to enzymatically hydrolyze intended parts of the polypeptide sequence [66].

Microorganisms’ resistance raises many challenges. The body’s antibiotic resistance is an important aspect that presents new challenges for the research domain. Besides the in vitro and in vivo investigations made until now, a new field is currently developing in silico. Digitalization principles are included progressively in biomedical sciences. Computer-based analytic methods can help researchers identify new mechanisms for CPPs in their scientific approach. Such techniques help create numerous theoretical models, helping with understanding the possible action patterns for microbial suppression [67].

The most projected interaction systems were considered the artificial phospholipidic bilayers. Sebastiao et al. used mammalian cells to create a double stratum. The main advantage of this approach is the similarity in action to biotic systems. The method is characterized by improved velocity. It analyzes the interferences determined on the lipid pores via fluorophore colocalization and FRET-based assays [68]. This technique is not limited to AP. It can be extended to all types of functional peptides.

### 2.2. Antimicrobial Activity

As previously mentioned, the antimicrobial activity of biologically active peptide depends on a large proportion of the environment acting conditions, such as pH [69]. Another important property is their hydrophobicity/hydrophilicity balance and/or the tridimensional structure. Hazam et al. started with two inactive peptides, tilapia piscidin 1 and 2. After modifying the electric charges, they obtained a new system, TP2-5 (KKCIAKAILKKAKKLLKKLVNP). It had antibacterial potential, the toxic response did not raise significant problems, and it was stable [70].

The *α*-helix form is considered to determine the peptide antimicrobial attribute. The property influence was highlighted in the Ma et al. investigation. They worked with histone1-derived antimicrobial peptides, LcH1-1 and LcH1-2 [67]. Additionally, several studies correlate the peptide’s spatial arrangement with the bactericide action due to their possible networking with the phospholipid layers [71,72]. The spiral shape varies, conditioned by the active component’s exhibit shape. In the case of hydrogels, it is *β* [73].

The peptidic sequence’s implication in the immune system’s function is known. Cai et al., in their recent research, evidenced the presence of a GzLEAP-2 amino acid sequence in different *Glyptothorax zanaensis* fish body components: liver, intestine, and muscles. The synthesized GzLEAP-2A and GzLEAP-2B peptides manifested antimicrobial activity upon encountering marine pathogens. They had 94 and 74 amino acid residues, respectively, and four and three cysteine rests, respectively [74]. The two peptides mentioned and the GzLEAP-2C expression with 83 amino acids revealed good results after testing the radical scavenging activities. The GzLEAP-2B and GzLEAP-2C elevated amounts exhibited anti-macrophage phagocytic properties.

The role in the dairy industry is known in the case of the *Lactobacillus acidophilus* bacteria. New elaborated investigations reveal potential significant properties of different common elements. Due to da Silva et al.’s research, the AMP production attribute has been outlined. The peptidic fraction of interest identified was named doderline. It has a molecular weight of 1788.01 Da and presents activity toward *Candida albicans* [72].

Another essential aspect of human health is the attention given to wound healing. For example, Zhou et al. designed a peptide hydrogel, Jelleine-1, with antibiotic function. It showed good biocompatibility in both in vitro and in vivo tests (against the following microorganisms: *Staphylococcus aureus*, *Escherichia coli*, *Candida albicans*, and *Methicillin-resistant Staphylococcus aureus*) [73].

Many AMPs are modeled based on a biotic model encountered in different animals/fishes [67,71,74,75,76] or plants [75,77]. The CaDef2.1G27-K44 peptide had an amino acid sequence detected in *Capsicum annuum* fruits as a model. Various substitutions determined a significant fungicide activity against *Candida* strains throughout cytoplasmatic solubilization [77]. The new peptides that were laboratory-synthesized proved to have good antimicrobial activity. HX-12C, formulated by Luo et al., has the same action pattern as the previous peptides. It generates the cell membrane permeabilization process [78]. The modifications made to the native peptide model have the role of improving the active molecule’s physical–chemical parameters. The main goals are to increase the specific reactivity, decrease the molecular weight, and improve the toxicological response. A peptide with a lower mass will have a higher capacity to break through the microbial cell membrane. The hydrophilic parameter has a significant role in the peptide action system. However, of the same importance is the molecule’s electric charge level. The destructuration degree is conditioned by this aspect [79]. Park et al., in their investigation, used lysin and alanine as substituents in the initial peptide structure. The new system acted against the bacterium configuration through lipopolysaccharide linkage, determining cell membrane perturbance [76].

However, in most cases, the AMP activity sums up just the microbicide aspect. There are examples in which these molecules double as modulators. In the case of the TroNKL-27 peptide, using gel retardation analysis, Zhang et al. determined its capacity to generate bacterial genomic DNA corruption [75].

### 2.3. Haemolysis Assay

An essential aspect of active membrane peptide design is their possible capacity to damage more than the cell of interest. In this regard, the red blood cells possess importance. The reaction conditions have a significant influence on establishing the peptide’s hemolytic particularities. The main aspects are related to the buffer and incubation time, the number of red blood cells (RBC) presented, anticoagulant type, etc. [80]. Based on testing different specific attributes of cation peptides, Horvat et al. propose that a possible drug carries minimal hemolytic negative effects on the buforin, Tat, penetratin, and Dhvar4 [80].

Various investigations emphasize the active peptide biomolecules encountered in dairy products. We name both the specific outcome and the auxiliaries used. It was previously mentioned that *Lactobacillus acidophilus* describes an AMP pattern. Tenore et al. characterized a peptide from Mozzarella di Bufala after in vitro gastrointestinal digestion. The protein they studied is vital to suppressing H_2_O_2_-induced human erythrocyte hemolysis [81]. Similar results have also been obtained regarding the seven peptides resulting from in silico lactalbumin hydrolysis [82]. Another study highlights the hydrolyzed peptides from camel milk protein’s protective role for erythrocytes. The anti-hemolysis effect depends on the protease used and the action time [83].

Edwards et al.’s investigations of two peptides, poly-arginine-18 (R18: 18-mer of L-arginine) and the D-enantiomer R18D, led to the discovery that these could be considered for possible further use. Besides AMPs, peptides are also studied as neuroprotective active molecules. It was found that they do not promote primary HCMCs degranulation or cause serious RBC hemolysis. The system, including R18, R18D, TAT-NR2B9c, and protamine, has less probability of determining histamine-mediated anaphylactoid reactions or hemolysis in case of IV fluids dispassion [84].

Aurein peptides have good prospects for potential applications in the pharmacology area. Some research has focused on improving their characteristics. The system that binds polyglycerol to aurein and its spin-off products (PEGylated aurein 2.1, aurein 2.6, and aurein 3.1) exhibits a decrease in antibacterial and hemolytic activity [85]. Such results increase their probability of being considered candidates for different medical applications.

New trends in research have as a common point the aim of developing structures similar to the natural ones. Mimetic systems are also gaining ground in the active peptides domain. Thus, the five tetrasubstituted, cationic, and amphipathic heterocycles were proposed as alternatives for AMPs. The structures considered presented insignificant hemolytic profiles [86]. Zou et al. used a similar approach to determine the viable active core. The proposed palustrin-2LTb system and its fractions were tested in vitro for anti-hemolytic activity using horse erythrocytes. Eliminating the threonine C-terminal element resulted in high activeness. The result was the contrary, continuing the suppressing process [87].

Bhat et al. designed an active peptic cationic amphipathic molecule suitable for application in aqueous environments. The simple KK16 structure they proposed displays weak hemolytic activity towards fish RBCs even at increased concentrations. The result followed the prediction made based on the in silico analysis. It did not predict such an effect [88].

### 2.4. Cell Uptake Assays

Another important characteristic of MAPs is their capacity to be cell-assimilated. The amino acids present in the peptidic sequence influence their properties. The availability of a cysteine rest increases cellular absorption in the case of CyLoP-1. It could be successfully used in human, animal, or plant pharmacology areas [89].

The Iztli peptides (IPs) belong to the hunter–killer peptides (HKPs) group. They both have the capacity to bind and are bacteriocidal. Two chromophores were used, TAMRA and Hilytefluor 488, to evidence the presence of IP-1 in the cell. In case of high membrane potential, IP-1 can create extensive passages for ion transfer. Their good result for penetrating animal cells is coupled with a significant toxicity level. The situation may be caused by the specific catcher needed for the precise targets [90].

Many active membrane peptides used as sources or models for determinations had a vertebrate pattern. A new modeling domain takes on a unique shape: ants. Ascoët et al. investigated the properties of two main linear α-helical polycationic peptides, bicarinalin (M-Tb1a) and U9-MYRTX-Tb1a (U9). Both cause membrane aperture, with the second one also affecting the mitochondria. The effect was observed at increased peptide levels [91].

Promising results are obtained for the peptidic structures synthetically generated. Configurations L-Trp-L-His(1-biphenyl)-NHBzl (12e) and L-His [1-(4-n-butylphenyl)]-L-Trp-L-His[1-(4-n-butylphenyl)]- NHBzl (16d), both solid and colorless, had the most significant attributes among the versions tested in the investigation made by Sharma et al. The initially considered structures were of di- and tripeptides. An essential factor in ensuring the structure’s hydrophobicity was the presence of the tryptophan amino acid. The results were obtained after in vitro determinations. The linkage to the cryptococcal cell layers resulted from non-polar interactions; these determined orifice formations. The TEM analysis revealed the peptide’s perturbance effect on fungal intracellular constituents [92].

An approach that may be the basis for further developments in active peptides is the use of composites. In the case of Wade et al., mixtures of four peptides were used, namely the translocating peptides, buforin II and DesHDAP1; the membrane lysis magainin 2; and parasin. The solubilization peptide type exhibited the principal role in the hybrid aggregate, with increased activity at the N-terminal atom [93]. Promising results have also been reported by Tang et al. They obtained the chimeric peptides P3I7 and P3L7 from the native swine antimicrobial peptide PR-39, and the mix of cationic cell-penetrating peptide (R6) with antimicrobial fragments changed with hydrophobic residues [94]. Such a perspective could provide a starting point for finding natural mixed compositions of peptides with various roles. Floral nectar represents a possibility. In the case of tobacco bloom, it was shown that the selected peptidic structures interacted with *Aspergillus niger* and *Escherichia coli* cells. This conclusion resulted from using fluorophore-reactive propidium iodide (PI). Its presence in the tested configurations confirms that the membrane was degraded, with the specific mechanism not being elucidated [95].

Efficient perspectives are also offered regarding the nanocomposite. The peptide–silver nanoparticles (P-AgNPs) proved to have increased stability and a good performance against *Pseudomonas aeruginosa* and *Staphylococcus aureus* cells. They could damage the peptidoglycan structure and discharge the AgNPs into the cells [96].

### 2.5. Vesicle Fusion and/or Leakage Assay

The efficiency of MAPs is usually studied through fluorescents. Wichmann et al. tested the possibility of using a flow cytometric assay to obtain extensive information regarding the MAP interaction. They were tagged with a specific fluorophore group and microscopically analyzed. Double-marked pores were utilized as patterns. To this end, extensive vesicles were designed. They enclosed 1 mol% of DOPE jointed to Atto655 as membrane label, 20 mol% of the negatively charged POPS to set the negative membrane similarly to the biological membranes, and 79 mol%. Using the system developed created the possibility of differentiating the vesicle action mechanisms [97]. Different investigations focus on developing simple, accurate, and real-time methods to determine the MAP’s effects on targeted cells. Sun et al. successfully designed a system that offered valuable information regarding the peptide’s lysis impact on free and immobilized cell patterns, as the carrier was considered the TAT system (YGRKKRRQRRR). It has 86 amino acids and can shift across the plasma layer, find the core, and be implicated in HIV reproduction. The research was based on the photo-voltage transient method, a dynamic giant bilayer vesicle (GBV) leakage method, and a giant unilamellar vesicle (GUV) leakage assay. The pH value was neutral, and there was a decreased ionic potential [98].

Possible agents to change the cell’s normal metabolism are the merged peptides. The results obtained by Basso et al. underline the efficiency of the internal fusion peptides, cIFPs. They can determine allosteric modifications of the viricidal proteins. The possible action mechanism may be based on cellular and endosome layer interference. The activity might be determined by the multiple GxxxG-like motifs from its first configuration [99]. Similar significant results were highlighted in the case of the cyclic peptides. They proved to significantly function in the layer fusion transformation of the lipid media characteristics [100]. Cummings et al. characterized the cryptdin-4 peptide as having properties to generate a special layer microstructure. Their shape was that of hemifusion vesicle aggregates. The generation process depends on different parameters, such as cell charge [101], composition [102,103], or the presence of other ions. A limited millimolar quantity of Ca^2+^ endorsed the enlargement of *β* shape [104]. The charge balance strongly influences the linking process between the peptidic and cell lipidic chains. There is a need for several steps to make the connections definitively. Other important aspects are the lipid vesicle aggregation status, gel or liquid, and the addition sequence. The polypeptides quickly crossed the POPG, the DPPG, and their 50% mix with phosphatidylcholines of equivalent chain length in the fluid phase. Also, the L-arginine polymers easily carry over the lipidic membrane, compared to the poly-L-lysines [101].

As mentioned, the peptide–target molecule interrelation is conditioned by several factors. Chiou et al. mentioned the importance of the cellular lipid sequence saturation and wrapping degree. The observation result may be due to the switch to layer micellization instead of penetration in case of a decreased cell bundling level [105].

### 2.6. Microfluidics

MAP analysis through microfluidics techniques is currently of great interest due to the possibility of working with small target molecule quantities [106]. Xun et al. successfully utilized flow-focused microchannel chips and ionic gelatinization technology to obtain capsules with silkworm pupae bioactive peptides. The CA-g-Ch-sodium alginate formula presented excellent resistivity to simulated digestion conditions [107].

Developing a small integrated system for different specific biological activities demands a broad view of all processes. The efficiency and high fidelity of the response are two important features. Nahas et al. designed an integrated lab-on-a-chip multilayer microfluidic platform. In the initial test phase, one current approach uses peptidic mimetic structure to test the specific properties, especially from the hemolytic and cell lysis point of view. This testing technique has a biomimetic design. It determines the formation of giant unilamellar vesicles (GUVs) in physiologically relevant buffers. The GUVs are immobilized separately to speed up the biocide distribution [108].

Also important in the biotechnology research domain are the cost and ease of use. Using passive micromixers helps screen non-rational libraries of MAPs expressed on yeast surfaces. Microfluidic stands can be suitable in the process of obtaining GUVs and as interplay environments [109]. Time efficiency is another aspect that currently has to be considered. Implementing the microfluidic platforms concept allows the testing of a significant number of molecules of interest simultaneously, ensuring comparable conditions [110]. The possibility of investigating at the same time an increased number of MAP in the presence of different possible target cells makes it easy to form links between molecular mechanisms of the action pattern. According to Nahas et al., investigations using a parallelized microfluidic platform for GUV sequestration can have single or multiple molecular mechanisms. For various processes exhibited, disturbance will result from the provocative type [111].

## 3. Biomedical Application

A recent review by Moretta et al. that presents the biomedical applications of AMPs, in which the antimicrobial activity of these peptides against different pathogens that are associated with human diseases was emphasized, was published [112]. The beneficial roles that numerous AMPs play in the treatment of skin infections and in the treatment of respiratory diseases and disorders caused by oxidative stress, such as obesity, diabetes, and chronic inflammatory intestinal disorders, were exhibited [112]. In point of fact, AMPs have the ability to regulate proinflammatory reactions, stimulate cell proliferation, and promote wound healing by modulating cell migration, angiogenesis, chemotaxis, and cytokine production. Regarding the anti-inflammatory, antibacterial, or drug delivery capacities of CPPs against carcinoma, both in vivo and in vitro tests were reported [30]. These are only some of the potential uses that might be found in the field of medicine, and complementary data are presented below.

### 3.1. Antibacterial

Antibacterial AMPs are the most studied AMPs to date due to most of them being cationic, which makes them target bacterial cell membranes and causes the disintegration of the lipid bilayer structure. Most of these AMPs are also amphipathic, with hydrophilic and hydrophobic domains. Such structures provide AMPs the capability to bind to lipid components (hydrophobic region) and phospholipid groups (hydrophilic region) [5,113]. It is interesting to note that studies have shown that certain AMPs can kill bacteria at low doses without affecting the integrity of the membrane. These AMPs kill bacteria by inhibiting several critical intracellular activities, including protein synthesis and DNA replication, rather than directly interacting with the membrane. Buforin II, for instance, may enter cells and bind to DNA and RNA without affecting the cell membrane. Other examples of these AMPs include drosocin, pyrrhocoricin, and apidaecin. It has been demonstrated that some AMPs can kill bacteria that are resistant to antibiotics. For instance, both the antibiotic vancomycin and the AMP nisin (Table 2) can prevent the formation of cell walls. Although a methicillin-resistant *Staphylococcus aureus* strain has been reported to be vancomycin-resistant, studies show it is still sensitive to nisin [113]. Here are some examples of the uses of peptides in different branches of the health industry.

#### 3.1.1. Systemic Infections

Peptides have primarily been used to treat bacterial skin infections, wounds, or pink eye. Examples include Neosporin^®^ (gramicidin; manufactured by Monarch Pharmaceuticals, Inc., Bristol, TN, USA), a triple antibiotic ointment for treating bacterial conjunctivitis; Vancocin^®^HCl (vancomycin; manufactured by ANI Pharmaceuticals, Inc., Baudette, MN, USA), used for the treatment of Gram-positive bacterial infections; Cubicin^®^ (daptomycin; manufactured by Merck & Co., Inc., Kenilworth, NJ, USA), which can be directly injected into the body and is an antibiotic used for the treatment of complicated skin infections and *Staphylococcus aureus* infections; Orbactiv^®^ (telavancin; manufactured by Theravance Biopharma, Inc., San Francisco, CA, USA), used for the treatment of Gram-positive bacterial infections [22,53]. Due to their longer elimination half-lives (which can range from hours to days) and improved pharmacokinetics when compared to gramicidin or other AMPs, several AMPs, including Cubicin, Vancocin, Orbactiv, Dalvance, Vibativ, and Coly-Mycins, have been licensed for direct injection into the human body [22].

#### 3.1.2. Food Preservation

To meet the rising demand from consumers for safe, ready-to-eat foods with minimal processing, bacteriocins (AMPs that kill bacteria), natural antibacterial agents, are desirable substitutes for chemical preservatives. Bacteriocins can be added to food products without altering their organoleptic characteristics because they are flavorless, odorless, and tasteless. Moreover, many bacteriocins maintain stability at various salt concentrations, high temperatures, and low pH values. Bacteriocins as food preservatives have multiple advantages: they increase food shelf life, provide additional protection during temperature misuse circumstances and at other crucial control points, reduce the danger of foodborne pathogen transmission via the food chain, and allow for less harsh treatments during food production without affecting food safety. As the only bacteriocin with a biopreservative license, nisin (Table 2) is used in various commercial preparations, including for Nisaplin^®^, Chrisin^®^, and Delvo^®^Nis. It is commonly used in the dairy industry to manage contamination from *Listeria* strains and *Clostridium* [114].

#### 3.1.3. Oral Care

Generally, bacteria produced by lactic acid form proteinaceous antibacterial metabolites with bactericidal or bacteriostatic activity against genetically similar bacteria. *P. pentosaceus* ST58 genome research revealed operons encoding for the bacteriocins pediocin PA-1 and Penocin A. *Listeria monocytogenes*, *Enterococcus* species, and several *Lactobacillus* species employed to characterize the activity spectrum were all inhibited by the generated bacteriocins [115]. In addition, *L. lactis* 27 has the most significant antibacterial efficacy against *S. aureus* CECT 239. Also effective at inhibiting many species of the genera *Enterococcus*, *Lactobacillus*, *Pseudomonas*, *Streptococcus*, *Staphylococcus*, and *Listeria* were the antimicrobials generated by *L. plantarum* ST16 Pa. In a related investigation, *B. lactis* CFS showed its antibacterial activity against both Gram-positive and Gram-negative bacteria by being able to reduce the growth of both *L. monocytogenes* and *E. coli* ATCC 25922 [116]. Overall, AMPs have considerable biotechnological potential for creating efficient bio-preservation cultures and potential therapeutic bacteria for human health [115].

### 3.2. Anticancer

Due to their high binding affinity, compact size, selective uptake, excellent durability, quick clearance from non-specific targets, and retainment in specific targets, the use of CPPs is a promising approach for tumor imaging [117]. CPPs can carry, convey, and deliver imaging agents, enabling intracellular functioning and access to the imaging cargo. To image tumors, CPPs/THPs can be coupled to radioisotopes, nanoparticles, fluorophore-labeled or activatable probes, quantum dots, polymers, metal chelates, and other contrast compounds [117,118].

Since THPs can target cancers with distinct lineages in vivo, these compounds are predicted to be suitable molecules for advanced anticancer therapies. The primary uses for THPs would be in vivo tumor imaging and therapeutics [118]. A peptide–drug conjugate consists of a THP (carrier), a protease-cleavable linker (linker), and an anticancer drug (payload). The peptide’s design can vary based on the chemical properties of the carrier. To achieve the best performance as a targeting tool, the peptide can vary in length, amino acid modification, and the use of d-amino acids, and the peptide might take a linear or circular form for stability in vivo [118].

By targeting cancer cells specifically, some bacteriocins have also demonstrated anticancer properties. Bacteriocins produced by Gram-positive bacteria, such as nisin, or by Gram-negative bacteria, such as microcin E492 (Table 2) and colicins (A, D, E1, E2, and E3), have shown lethal effects on malignant human cell lines [114,119]. Bacteriocins react against cancer cells by inducing apoptosis and/or depolarizing the cell membrane, affecting the cell’s permeability [119]. For instance, it was demonstrated that nisin promoted DNA fragmentation or apoptosis and decreased cell proliferation by inducing cell cycle arrest in head and neck squamous cell carcinoma cells. Another example is the bacteriocin called azurin, which is produced by a strain of *P. aeruginosa* and was investigated as a potential anticancer medicine due to its preferential attachment to human cancer cells and subsequent cytotoxic and apoptotic effects, which did not appear to harm normal cells. However, most investigations of the anticancer activities of bacteriocins have been conducted in vitro, emphasizing the need for in vivo validation [114].

Many peptides are undergoing different clinical trial phases or have already been approved by the FDA. Below, we will look further into some relevant examples.

#### 3.2.1. Peptides Used for Lung Cancer Treatment

Many years ago, a peptide was utilized as a lung cancer predictor. New peptides have recently proven to be effective at identifying lung cancer. Accordingly, studies have shown that non-small-cell lung cancer patients have greater levels of the linear peptide antigen generated from annexin A1 than control subjects [120]. Moreover, individuals with lung cancer had increased serum levels of C-peptide, particularly those with small-cell, stage III-IV lung cancer. Interestingly, peptides could be used as carriers due to their particular high affinity. Peptides are also effective in the treatment of lung cancer. For instance, in studies, the synthetic peptide Disruptin reduced the capacity of EGFR-dependent cancer cells to proliferate and clone. Also, it was observed that Disruptin could reduce lung cancer cell line microvessel density in H1975 xenografts. The peptide isolates from high oleic acid soybean exhibited a dose-dependent inhibitory effect on cancer cells. Furthermore, peptides extracted from the venom of the Eastern green mamba are cytotoxic to the human NSCLC cell line A549 [121].

#### 3.2.2. Peptide Treatment for Multiple Myeloma Tumors

To treat myeloma and amyloid light chain amyloidosis, Oncopeptides AB developed Melflufen 1, an ester of an alkylated dipeptide. This drug is used for the early treatment of refractory multiple myeloma [122]. Melflufen targets the processes of numerous myeloma malignant transformations with a special mechanism that makes it a member of a new class of peptidase-enhancing drugs. Melflufen’s activity is achieved by aminopeptidase via a precise peptide link, leading to a peptidase augmentation effect. Following positive research, the US Food and Drug Administration (FDA) rushed the approval of Melflufen in 2021 to treat three different kinds of refractory multiple myeloma [122]. However, in October of the same year, due to the phase III clinical trial’s failure to successfully lower the risk of death in patients with relapsed refractory multiple myeloma, Oncopeptides AB decided to remove Melflufen from the US market [122].

#### 3.2.3. Peptide Treatment for Neuroendocrine Cancer

Lu-DOTATATE is a peptide-based drug containing somatostatin as a homing peptide conjugate with a radio-therapeutic agent [123]. It treats gastroenteropancreatic neuroendocrine tumors that develop within tumor cells and kill them with cytotoxic radiation [123,124]. A recent randomized controlled clinical experiment assessed Lu-DOTATATE’s effectiveness and safety. They discovered that Lu-DOTATATE considerably increased response rates and prolonged disease-free survival, reducing the symptoms of hormone over-secretion. Consequently, Lu-DOTATATE may be a valuable and successful treatment for neuroendocrine cancer; however, there have not been many systematic reviews and meta-analyses to determine its benefits. A control ratio of about 78–79% demonstrates the remarkable efficacy of the Lu-DOTATATE therapy. Moreover, Lu-DOTATATE’s side effects, such as exhaustion, nausea, vomiting, and hormone imbalances, were barely perceptible [124]. Because of that, this medicine containing a somatostatin analog is FDA-approved [123].

#### 3.2.4. Peptide-Based Drugs for Ovarian Cancer

The current primary ovarian cancer care standard is adequate debulking surgery, iv carboplatin/cisplatin, and paclitaxel-based chemotherapy. Recently, the peptide-based medication Melflufen, which was previously mentioned in this review as being used for myeloma patients, was discovered to be active against ovarian cancer cell lines and primary cultures of patient-derived ovarian cancer cells. It also inhibited the growth of subcutaneous A2780 ovarian cancer xenografts when administered alone, in combination with gemcitabine, or with liposomal doxorubicin when given intravenously [125]. Also, a sub- and intraperitoneal xenograft model demonstrated the effectiveness of intraperitoneally administered Melflufen for treating peritoneal carcinomatosis while causing only moderate systemic exposure and few adverse effects [126].

Paclitaxel is also undergoing different research studies for using this drug in other types of cancer, such as brain tumors or breast cancer [123].

#### 3.2.5. Peptide-Based Drugs Used for the Treatment of Prostate Cancer

The gastrin-releasing peptide receptor is a promising molecular target for prostate cancer imaging and treatment. Bombesin peptides have a high affinity for this receptor and can be used for imaging and therapy. Bombesin is a 14-amino-acid peptide agonist, and several analogs based on this 14-amino-acid backbone have been studied as diagnostic and therapeutic targeted agents [127]. Another approach in prostate cancer treatment uses PROTAC. The proteolysis-targeting chimera (PROTAC) drug design approach presents a novel possibility for developing cancer therapies involving the induction of protein degradation. A PROTAC medication is a heterobifunctional compound with two ligands—one for an E3 ligase and one for the target protein of interest. Au-AR pep PROTAC fully inhibits the development of AR-positive prostate cancer cells at high concentrations (above 500 nM). The fact that Au-AR pep-PROTAC inhibits AR-positive cancer cells suggests that AR is rapidly destroyed at the protein level, as other small molecules that target AR have similarly demonstrated total suppression of AR-positive cancer cells [123].

### 3.3. Vaccines

Peptide vaccines have attracted attention in the immunotherapy of several cancers, including breast and lung cancer, but also in treating viral diseases, including human papillomavirus, influenza, and HIV, and neurological disorders, including Alzheimer’s [2]. The primary objective of peptide vaccines is to create T-cell and B-cell epitopes capable of inducing specific immune reactions while immunodominant [128,129]. To facilitate the ability of the peptide vaccines to pass the membrane, emulsion administration is usually performed [2]. The vaccines listed below are only a few peptide-based vaccines now undergoing clinical trials.

#### 3.3.1. Antitumoral Vaccines

Due to peptides’ chemical stability and well-defined structure, they have a variety of benefits over conventional antitumor immunotherapy techniques. No contagious diseases or contaminated materials are involved in their production, so they are simple and inexpensive. Synthetic peptides can be combined with various substances and T-cell epitopes to increase their immunogenicity. For cancer immunotherapy, several vaccines based on various peptides have been studied. Below are some peptide vaccines that have undergone phase I, phase II, or phase III clinical studies. [129].

#### Heregulin (HER)-2/neu Peptide

The 185 kDa transmembrane protein HER-2/neu belongs to the epidermal growth factor receptors family. On the epithelial surface of healthy tissue, HER2/neu is only moderately expressed, but it is overexpressed on the surfaces of breast, ovarian, and lung tumor cells. Because HER2/neu is overexpressed in about 30% of patients with breast cancer, it makes an intriguing target for immunotherapy. A monoclonal antibody against HER-2/neu called “Herceptin” has been approved for breast cancer treatment [129].

The mucin-1 (MUC-1) peptide is a glycoprotein belonging to the mucin family that has a polypeptide core with a variable number of tandem repeats (VNTR) of a 20-amino-acid sequence. Recent studies show that MUC1 is implicated in tumorigenesis, tumor cell movement, resistance to chemotherapeutic drugs, and stress-induced apoptosis. In individuals with advanced adenocarcinoma, high serum MUC1 levels are linked to immunosuppression and a poor prognosis [130]. MUC-1 glycosylation is abnormally reduced in carbohydrate side chains in malignancy. The immune system’s exposure to MUC1 peptide epitopes increases as a result. These epitopes can induce antibody and cytotoxic T-cell responses [129]. A cancer vaccine known as the BLP25 liposome vaccine (L-BLP25) targets the exposed core peptide of MUC1 tumor-associated antigen. The 25-amino-acid sequence of the BLP25 lipopeptide confers MUC1 affinity. A multinational, randomized phase III trial of the L-BLP25 vaccination against placebo in patients with stage III NSCLC has commenced in response to the promising findings of the randomized phase II trial [130].

#### 3.3.2. Antiviral Vaccines

Effective vaccines are still not attainable for many infectious diseases, despite vaccines’ critical role in the fight against viral infections. The significance of vaccines during the onset of a pandemic, such as the COVID-19 pandemic, cannot be understated. The toolkits available for modern vaccine development have been widened by our growing understanding of genetics, structural biology, and innate/adaptive immunity [131].

*HPV vaccine*: Antigens encoded by viral genomes can be found in many malignancies. Several tumors are caused by viruses, including B-cell lymphomas linked to the Epstein-Barr virus (EBV), adult T-cell leukemia linked to the human T lymphocyte-1 (HTL-1), and cervical cancer linked to the human papillomavirus (HPV). HPV is thought to be the primary cause of more than 90% of human cervical cancers, and HPV DNA has also been found in other tumors, such as penile and pharyngeal carcinoma [129]. Several synthetic peptide vaccines, including PepCan and ISA101, that target the HPV 16 E6 and E7 proteins have displayed regressions in cervical and vulvar high-grade intraepithelial lesions. Patients with metastatic or recurrent oropharyngeal cancer that is HPV 16-positive are the subjects of a phase II investigation focusing on ISA101b. Moreover, DPX-E7, a peptide vaccine, is undergoing phase I/II studies to treat incurable head and neck malignancies [132].

*Influenza and HIV-1 vaccines:* Due to their extreme sequence variety, the influenza virus and human immunodeficiency virus type 1 (HIV-1) have proven to be two of the most problematic viruses for vaccine development [133].

Epitopes from influenza proteins recognized by B- and T-cells are synthesized to create influenza peptide-based vaccines. The peptides are deposited onto liposomes or virosomes, which convey the antigen to the desired cells. In some vaccine types, peptide-based epitopes from nucleoprotein, hemaglutinin, and matrix 1 protein target B- and T-cells. In contrast, other vaccine types use in silico multiple alignments of influenza sequences to suggest epitopes that would make suitable candidates for vaccine production. Clinical trials for these vaccines are currently in phases 1 and 2, and the results for both humoral and cellular immunity are encouraging [134]. Multimeric-001, the most advanced influenza peptide vaccine, combines nucleoprotein, matrix 1, and both B- and T-cell linear epitopes from HA into a single recombinantly synthesized polypeptide [133].

The Rv144 clinical trial for HIV-1 revealed that the V3 loop of gp120 targets neutralizes antibodies, which has sparked much interest in it as a potential target for peptide-based vaccine development. Synthetic V3 peptide has been produced using chemical or chemical/chemoenzymatic techniques in both mono- and diglycosylated forms. In addition, more recent studies have looked at synthesizing multicomponent and multivalent V3 glycopeptides [133].

*COVID-19 vaccine:* EpiVacCorona is a peptide-based vaccine manufactured by the Russian biological research center “Vector Institute.” The vaccine is focused on the use of snippets of synthetic viral peptides, including one spike, one protein N, and one bacterial peptide, which are coupled to a large carrier protein representing SARS-CoV-2 antigens and adjuvanted with aluminum hydroxide [135,136]. As protein N was discovered to trigger T-cell immune responses, it was theorized that it might be an important component of future SARS-CoV-2 vaccines. Protein N, a rich source of CD4+ T-cell epitopes in the EpiVacCorona vaccine, was chosen to serve as a carrier for peptide antigens due to its high level of immunogenicity and conservation [136]. Even though, in the 21 days following the second injection, 100% of the volunteers were found to have a seroconversion with a neutralizing antibody titer of 1:20, recent research indicates that EpiVacCorona is an ineffective vaccine that will not shield against COVID-19 [135,136].

#### 3.3.3. Vaccines for Neurodegenerative Diseases

Antigen-presenting cells use specialized receptors to recognize foreign antigens that have entered the human body to initiate potential peptide-based vaccines’ mechanisms of action for neurological diseases. For antigens to be expressed on the surface of antigen-presenting cells as peptide epitopes in the major histocompatibility complex (MHC) class II, a peptide–MHC II complex must first be ingrained into antigen-presenting cells via receptor-mediated phagocytosis or endocytosis and processed by the proteasome. Then, using specialized receptors that identify peptide–MHC II complexes, T helper cells communicate with antigen-presenting cells. These situations cause CD4+ T-cells to become activated, enabling them to engage with B-cells and promote antibody production [137].

The pathologic neurodegeneration that is the defining feature of Alzheimer’s disease is supported by two proteins, Aβ and Tau, which aggregate abnormally and play a significant part in this process [133,137]. Hence, the idea that immunization with epitopes from these proteins might lead to preventive clearance of their neurotoxic forms or prevent the formation of aggregates entirely is of great interest. However, breaking through the blood–brain barrier, typically inaccessible to antibodies, presents an obstacle for this method. Nonetheless, some peptide-based vaccine candidates have advanced to clinical testing, demonstrating that specific immune responses may prevent aggregates from building up in the brain [133].

*Vaccine studies for dementia:* Based on the well-known “amyloid cascade hypothesis,” neurotoxic Aβ may generally be formed under specific conditions, accumulate or aggregate, and eventually affect neurons, resulting in dementia. The hypothesis that antibodies targeting Aβ may disrupt its aggregation and, thus, limit the evolution of the disease is the basis for the idea that immunization against Aβ is an active immunotherapy for dementia or Alzheimer’s. However, one should remember that Aβ is a self-peptide that could either be non-immunogenic or cause a harmful autoimmune reaction; as a result, the effectiveness and safety of active immunotherapies are critical issues that need to be addressed [138].

Most peptide-based vaccines now being developed target specific peptide epitopes of biomolecular targets linked to the onset/progression of neurodegeneration, such as polypeptide Aβ or the proteins tau and α-syn. Certain variables, like the precise peptide epitope and the formulation components utilized, significantly impact peptide-based vaccines’ overall efficacy and safety. Additional developments (e.g., identifying novel biomolecular targets associated with neurodegeneration and improving formulation techniques) are anticipated to speed up/facilitate research in this specific field of immunotherapy with a view to its eventual clinical application [138].

#### 3.3.4. Arginine-Rich Peptides as Neuroprotectants in Ischemic Stroke

In the last decade, investigations found that arginine-rich cationic CPPs are neuroprotective in in vitro neuronal cell stroke models. TAT and other neuroprotective peptides coupled to CPPs are effective in various acute neurodegenerative diseases such as traumatic brain injury, stroke, and prenatal hypoxia–ischemia [139]. One of the primary mechanisms triggering neuronal degeneration in stroke is known to be excessive glutamate release from synaptic junctions and related cytotoxicity [140,141]. Studies show that the polyarginine ST2-104 peptide may prevent glutamate-induced cell death in SH-SY5Y neuroblastoma cells, modulate calcium intake, and avoid apoptosis and autophagy. It is also known that the arginine-rich R18 peptides (polyarginine-18, L-isomer, and D-isomer) boost the optimum activity of the thrombolysis response while preserving their neuroprotective qualities when co-administered with thrombolytic drugs [140]. While it is undoubtedly true that the arginine content of the peptide is significant in this regard, it is also notable that other amino acid residues can affect peptide behavior. This ability could be used to target and modulate the activity of particular neuronal and non-neuronal receptors inside the central nervous system for the research into and development of therapies for additional neurological conditions (such as epilepsy and multiple sclerosis) [139]. Likewise, arginine-rich peptides act as oxidative stress reducers and scavengers of harmful compounds. Moreover, they possess anti-inflammatory, antibacterial, and anti-cancer effects. By working as a reliable method of delivering nucleic acids, arginine-rich peptides have the potential to be fundamental in advancing several sectors, such as gene therapy, imaging, and gene vaccines [141].

**Table 2:** Peptides with applications in biomedicine.

Peptide	Commercial Drug Name	Applications	Trial Phase	References
Antibacterial				
Gramicidin	Neosporin^®^	Treating bacterial conjunctivitis	Admitted to the market	[22]
Vancomycin	Vancocin^®^HCl	Treatment of Gram-positive bacterial infections	Admitted to the market	[22]
Daptomycin	Cubicin^®^	Treatment for skin infections and *Staphylococcus aureus* infections	Admitted to the market	[22]
Telavancin	Orbactiv^®^	Treatment of Gram-positive bacterial infections	Admitted to the market	[22]
Nisin	Nisaplin^®^, Chrisin^®^ and Delvo^®^Nis	Biopreservative	Admitted to the market	[114]
*Streptococcus salivarius* K12	BLIS K12	Protection against oral pathogenic bacteria in humans	Admitted to the market	[142]
Antitumoral				
Microcin E492	-	Cervical adenocarcinoma, acute T-cell leukemia, Burkitt’s lymphoma, B-lymphoblastoid cells	Preclinical	[143,144]
Colicin A and E1	-	Breast carcinoma, osteosarcoma, leiomyosarcoma, fibrosarcoma	Preclinical	[143,144]
Azurin-Derived Peptide p28	-	Breast cancer	Phase I of clinical trials	[145]
Nisin A	-	Head and neck squamous cell carcinoma, breast adenocarcinoma, liver hepatocellular carcinoma, acute T-cell leukemia	Preclinical	[143,144]
Pediocin CP2	-	Mammary gland adenocarcinoma, hepatocarcinoma, cervical adenocarcinoma	Preclinical	[143,144]
Peptide	Commercial Drug Name	Used for	Trial Phase	References
Vaccines				
Combination of 25 amino acids from several immune mutations of the repetitive region of MUC1 combined with immunoadjuvant monophosphoryl lipid A	Liposomal BLP-25	Anticancer	Phase III of clinical trials	[146]
Combination of 16 peptides	ISA101	HPV immunization	Phase II of clinical trials	[147]
Combination of nucleoprotein, matrix 1, and both B- and T-cell linear epitopes from HA into a single recombinantly synthesized polypeptide	Multimeric-001	Influenza and HIV-1 immunization	Phase I of clinical trials	[133]
Synthesized peptide immunogens of the SARS-CoV-2 S protein conjugated to a carrier protein and absorbed on aluminum hydroxide	EpiVacCorona	Coronavirus disease 19 (COVID-19) immunization	Phase II of clinical trials	[148,149]
Short C-terminal fragments of Aβ40 and an aluminum hydroxide adjuvant	ABvac40	Treatment for Alzheimer’s disease	Phase II of clinical trials	[150]

## 4. Current Limitations and Future Perspectives

Given their appealing pharmacological profile and underlying qualities, peptides are great starting points for developing new therapies [151,152]. Regarding their present limitations, naturally occurring peptides frequently are not ideal for treatments because of their physicochemical properties: metabolic instability (which leads to poor oral bioavailability), rapid clearance, high hydrophobic character, poor membrane permeability, and high manufacturing cost [152,153]. For them to be used in therapies, these issues must be solved. Through peptide structural modulation (backbone modifications, N-heterocyclic conjugation, modification of the N- or C-terminus by N-alkylation, amidation or esterification, cross-coupling reactions, and ligation strategies) and techniques including the replacement of natural amino acids with synthetic or modified amino acids, some of these issues have been addressed [152]. As Li et al. mentioned in their study, there are two main reasons for the low number of MAPs that have practical applications in the therapeutical field. It proved to be challenging to explain their exact action pathway fully. Another limitation is the need for more accurate concept projection [154]. Another factor that might explain the relatively slow rate of effective utilization of MAPs in practice is their deficiencies shown at the level of steadiness in the biomedical field. The amino acid’s specific characteristics may determine such constraints. Wang et al. evidenced around 80 peptides with curative properties that satisfied the strict regulations imposed on the therapeutic field in particular [3]. Also worth mentioning is the aspect highlighted by Kmeck et al., who sustained there is a limited possibility of using the MAPs in a unique therapeutic formulation. A viable solution could be the projection of a mutually reinforcing partnership between them and traditional and/or innovative pharmaceutical formulations [155] to overcome regular microbial resistance [20]. As a result, numerous peptide technologies, such as CPPs, peptide–drug conjugates, multifunctional peptides, and technologies emphasizing various administration methods, have been developing as promising alternatives to current therapies for emerging diseases like bacterial infections and tumor cells [156]. Since therapeutic candidates with dual-target pharmacology exhibit more complex in vivo outcomes than those with single-target pharmacology, producing multifunctional peptides may be challenging. Along with potential difficulties in translating results from in vitro to in vivo, applying findings from animal models to humans may be riskier for multifunctional peptides than for single-receptor peptides. Due to these factors, it is reasonable to assume that rather than entirely new peptide combinations, multifunctional peptides may develop primarily from established paradigms [151].

Future perspectives on peptide-based drug therapies suggest that more orally bioavailable peptides will likely be developed, as they are more patient-friendly [151,152]. Of course, the severity of the condition must be considered; for instance, in the case of diabetes, innovative approaches pursue the production of smaller needles or more practical devices. As for now, only a small number of oral peptide drugs are available, desmopressin (Minirin) and cyclosporine (Neoral) being two notable examples [151]. The poor metabolic stability of orally bioavailable peptides enquires frequent dosage. The acidic and enzymatic breakdown of the molecules in the gastrointestinal system is another one of the difficulties in developing oral peptides [153]. Peptides are often supplied parenterally to avoid first-pass metabolism for the reasons above [149,150]. Consequently, methods to develop oral administration peptides include stabilizing secondary structures such as stapled peptides, creating hydrophobic faces, cyclization, N-methylation, and creating intramolecular hydrogen bonds [151].

A recent article from Sharma et al. [150] states that 6% of all drugs approved by the FDA are peptides, whereas 83% are small molecules and 11% are biologics. However, the need for peptide therapies has grown significantly over time, resulting in the approval of over 100 peptide drugs [152]. FDA-approved drugs exhibited a decade-by-decade trend of consistently increasing approvals. These difficulties hampered the early years of the development of peptide-based medications through peptide synthesis and purification. As peptide therapies gained recognition, more and more peptide-based drugs were approved yearly, ranging from promising antibiotics to antidiabetic medicines and diagnostics agents [22]. Oral peptides are now virtually as prevalent in clinical trials as intravenous ones. Given that oral delivery is anticipated to expand the applicability of peptide medications, this is a promising course [152].

## 5. Conclusions

This paper presents an overview of membrane-active peptides (MAPs), covering their diverse physiological activities, such as antimicrobial, drug delivery, and cell-penetrating functions. It comprises several chapters that encompass the description of MAPs, their origins, synthesis methods, physicochemical properties, course of evolution, current applications, and future perspectives. MAPs, sourced from various organisms and synthesized using techniques like solid-phase peptide synthesis and recombinant DNA technology, target the lipid bilayer of cell membranes, exhibiting a broad spectrum of activity against bacteria, fungi, and cancer cells. Despite limitations such as enzymatic degradation and cytotoxicity, ongoing research focuses on peptide modifications, structural modulations, and developing delivery systems and nanostructures to enhance their stability, selectivity, and therapeutic applications. With advancements in these areas, membrane-active peptides are promising in antimicrobial therapy, drug delivery, gene therapy, and nanotechnology.

## Figures and Tables

**Table 1:** Physicochemical properties and origins of relevant membrane-active peptides.

Membrane-Active Peptides	Peptide Sequence	Aminoacids	Net Charge (at pH = 7)	Hydrophobicity (%)	Peptide Origin
AMPs					
α-Defensin	DCYCRIPACIAGERRYGTCIYQGRLWAFCC	30	1.73	23	Human
BuCATHL4C (CAT)	RIRFPWPWRWPWWRRVRG	18	5	33	Buffalo
Indolicin	ILPWKWPWWPWRR-NH_2_	13	4	23	Cow
Lactoferricin B	KCRRWQWRMKKLGAPSITCV	20	5.91	40	Cow milk
Piscidin 3	FFHHIFRGIVHVGKTIHRLVTG	22	3.36	14	Fish
Parasin	KGRGKQGKVRAKAKTRSS	18	8	61	Fish
Drosocin	GKPRPTSPRPTSHPRPIRV-NH_2_	19	6.09	37	Fly
Magainin 1	GIGKFLHSAGKFGKAFVGEIMKS	23	3.09	30	Frog
Histatin 5	DSHAKRHHGYKRKFHEKHHSHRGY	24	5.63	46	Human
Cathelicidin	HLLGDFFRKSKEKIGKEFKRIVQRIKDFLRNLVPRTES	37	6	54	Humans and other mammals
Protegrin-1	RGGRLC_1_YC_2_RRRFC_2_VC_1_VGR-NH_2_	18	6.82	33	Pig
PR-39	RRRPRPPYLPRPRPPPFFPPRLPPRIPPGFPPRFPPRFP	39	10	26	Pig
Cecropin B	KWKVFKKIEKMGRNIRNGIVKAGPAIAVLGEAKAL-NH_2_	35	8	37	The giant silkmoth, Hyalophora cecropia
CPPs					
TP10	AGYLLGKINLKALAALAKKIL	21	4	24	Analogue of transportan
Penetratin 1	RQIKIWFQNRRMKWKK-NH_2_	16	8	63	Fly
HIV-1 Tat	YGRKKRRQRRR	11	8	82	HIV infected cells
Bactenecin (Bac7)	RRIRPRPPRLPRPRPRPLPFPRPG	24	9	38	Mammals
Maurocalcine	GDCLPHLKLCKENKDCCSKKCKRRGTNIEKRCR	33	6.82	55	Scorpion
Transportan	GWTLNSAGYLLGKINLKALAALAKKIL-NH_2_	27	5	26	Scorpion venom
Nona-arginine (R9)	RRRRRRRRR	9	9	100	Synthetic
R6/W3	RRWWRRWRR	9	6	67	Synthetic
CyLoP-1	CRWRWKCCKK	10	4.86	50	Synthetic
CB5005	KLKLALALALAVQRKRQKLMP	21	6	38	Synthetic
Mastoparan	INLKALAALAKKIL-NH_2_	14	4	29	Wasp venom

Peptide sequences and origins were determined using the PubChem library [50], and the physicochemical properties were calculated using the BACHEM calculator (https://www.bachem.com/knowledge-center/peptide-calculator/, accessed on 25 April 2023).

## Data Availability

Not applicable.
